# CKD-MBD in Brazil: the gap between reality and the recommended guidelines

**DOI:** 10.1590/1678-4685-JBN-201800010003

**Published:** 2018-04-19

**Authors:** Melani Ribeiro Custódio

**Affiliations:** 1 Universidade de São Paulo, Faculdade de Medicina, São Paulo, SP, Brasil.

Disturbances in mineral metabolism and bone disease are common complications of chronic kidney disease (CKD), associated to a decreased quality of life and increased morbidity.

In 2006, the KDIGO changed the nomenclature for mineral metabolism abnormalities from renal osteodystrophy (ROD) to chronic kidney disease-mineral and bone disorder (CKD-MBD); ROD remained to describe bone morphology abnormalities.^1^ Since CKD-MBD is a systemic disorder, its treatment or prevention is fundamental to decrease the risk of severe complications such as cardiovascular disease (CVD), bone loss and fracture, inflammation and mortality.^2^ CVD is still the leading cause of death in uremic patients, and CKD-MBD consistently plays a central role in developing vascular calcifications. In addition, it contributes to the initiation or progression of left ventricular hypertrophy (LVH), in which fibroblast growth factor-23, a hormone produced by bone cells, has a pivotal participation.^3^

The greatest challenge in CKD treatment is preventing bone loss and fractures, which have a 4-fold increase in hemodialysis patients when compared to population-based controls in the United States. The high risk of fracture in these patients might be explained by abnormalities of biochemical and endocrine parameters, and the presence of uremic toxins that cause bone loss and deterioration of bone quality.^4^

In clinical practice, bone biopsy is not routinely used, as it is an invasive and often expensive procedure. In addition, the obtained samples require specialized processing that is not widely available. In the last years, serum biomarkers, mainly plasma levels of PTH, have been used as a surrogate indicator of bone turnover. Recently, serum phosphorus (P) has been correlated with bone turnover and the combined analysis with serum PTH provides valuable help in diagnosing ROD.^5^ In the absence of bone biopsy, PTH levels, in association with serum calcium (Ca), P, and alkaline phosphatase (AP) levels are useful to evaluate, diagnose, and guide the treatment of ROD.

Current strategies have focused on the use of PTH as a marker of bone turnover, and the treatment is mainly aimed at lowering PTH levels with vitamin D receptor activators or calcimimetics.^6^ However, a successful control of bone disease depends on several factors such as the country of origin, the characteristics of the dialysis population, resources for prevention and treatment of CKD-MBD, and access to new medications among others. Accordingly, it is important to be aware of these details, in order to establish strategies for mineral disease treatment.

In the paper entitled “Evaluation of prevalence, biochemical profile and drugs associated with chronic kidney disease mineral and bone disorder in 11 dialysis centers” the authors analyzed a sample of 1,134 patients on dialysis from different regions of a populous Brazilian state, Minas Gerais, as an example of the population and socioeconomic diversities of the country. According to the Brazilian dialysis census of 2016, we have more than 122,000 patients on dialysis, and CKD is considered a significant public health problem. Usually, nephrologists consider serum PTH, Ca, P, and total AP to guide the pharmacological treatment of CKD-MBD, as recommended by current guidelines.^7^ However, the above-mentioned manuscript has showed important points:

1. The authors divided patients into several ranges of serum PTH levels and confirmed previous data in which patients who have extreme levels, low or high PTH, exhibited higher levels of Ca and P, displaying a major risk for CVD and fractures;
2. PTH was outside the recommended target in 50.5% of the studied patients, and uncontrolled hyperphosphatemia was observed in 35.8% of patients. These results led to the conclusion that despite the knowledge of biochemical and demographic profiles of the dialysis population, it has been difficult to optimize treatment, most likely due to the lack of availability of some drugs. Therefore, authors have shown an excessive use of calcium-based phosphate binders (55.6%) to control hyperphosphatemia, instead of sevelamer (14.7%), and negligible use of medications to control PTH secretion such as receptor VD activators (0.2%) and calcimimetic drugs (3.5%). A concern was that even among patients with serum PTH bellow 150 pg/mL there was a significant proportion of patients receiving calcium carbonate phosphate binders (44.3%).
3. The observed low prevalence of CVD (11.8%) might be underestimated possibly due to the difficulty of complimentary tests;
4. It is difficult to compare the Brazilian results with those of other centers around the world. The intravenous paricalcitol was introduced in many countries in 1998 and oral paricaltitol in 2008. Cinacalcet hydrochloride was approved for use in Europe and in the US in 2004, and became clinically available in Japan in 2008.^8^ Combination therapy of active vitamin D analogues and cinacalcet hydrochloride dramatically improves the SHPT management and significantly decreases the number of parathyroidectomies. Unfortunately, these drugs began to be distributed to patients on dialysis by the public health network in Brazil only in 2018.

The above study reflects the reality of CKD-MBD treatment in Brazil and, as the authors mentioned, it is important that other similar studies be carried out to address the issue. Another key point is to convince government officials about the necessity and importance of improving public health policies regarding the treatment of CKD-MBD, based on epidemiology, guidelines, practices, and costs, therefore preventing CVD and fractures. This approach would certainly benefit the population on dialysis, improving quality of life and decreasing mortality.
